# App-based interventions for the prevention of postpartum depression: a systematic review and meta-analysis

**DOI:** 10.1186/s12884-023-05749-5

**Published:** 2023-06-14

**Authors:** Yumika Miura, Yusuke Ogawa, Ayako Shibata, Kyosuke Kamijo, Ken Joko, Takuya Aoki

**Affiliations:** 1 Hamamatsu Satocho Clinic, 1-22-22 Sato, Naka-Ku, Hamamatsu-Shi, Shizuoka 430-0807 Japan; 2 Department of Healthcare Epidemiology, School of Public Health, Kyoto University, Yoshida Konoe-Cho, Sakyo-Ku, Kyoto, 606-8501 Japan; 3 Department of Obstetrics and Gynecology, Yodogawa Christian Hospital, 1-7-50, Kunijima, Higashiyodogawa-Ku, Osaka, 533-0024 Japan; 4 Department of Gynecology, Nagano Municipal Hospital, 1333-1 Tomitake, Nagano, 381-8551 Japan; 5 Department of Obstetrics and Gynecology, Kikugawa General Hospital, 1632 Higashiyokochi, Kikugawa, Shizuoka 439-0022 Japan; 6 Division of Clinical Epidemiology, Research Center for Medical Sciences, The Jikei University School of Medicine, 3-25-8 Nishishimbashi, Minato-Ku, Tokyo, 105-8461 Japan; 7 Section of Clinical Epidemiology, Department of Community Medicine, Graduate School of Medicine, Kyoto University, 54 Kawahara-Cho, Syogoin, Sakyo-Ku, Kyoto, 606-8507 Japan

**Keywords:** Postpartum depression, Prevention, Application, Apps

## Abstract

**Background:**

This study explored whether psychosocial intervention applications (apps) are effective in preventing postpartum depression.

**Methods:**

We conducted an initial article search on 26 March 2020, and the updated search on 17 March 2023 on the electronic databases of the Cochrane Central Register of Controlled Trials (CENTRAL), MEDLINE via Ovid, Scopus, PsycINFO, CINAHL, and ProQuest Dissertations & Theses A&I. Furthermore, we searched the International Clinical Trials Platform Search Portal (ICTRP), and Clinical Trials.

**Results:**

We identified 2515 references, and sixteen studies were ultimately included in this review. We conducted a meta-analysis of two studies on the onset of postpartum depression. There were no significant differences between the intervention and control groups (RR 0.80; 95% CI 0.62 to 1.04; *P* = 0.570). We performed a meta-analysis of the Edinburgh Postnatal Depression Scale (EPDS). The intervention group had significantly lower EPDS scores than the control group (mean difference -0.96; 95% CI -1.44 to -0.48; *P* < 0.001, I2 = 82%, Chi^2^ = 62.75, *P* < 0.001; high heterogeneity).

**Conclusion:**

This study presents the results of current RCTs on interventions with apps, including an app with an automated psychosocial component for preventing postpartum depression that has been conducted. These apps improved the EPDS score; furthermore, they may prevent postpartum depression.

**Supplementary Information:**

The online version contains supplementary material available at 10.1186/s12884-023-05749-5.

## Background

Postpartum depression, in clinical practice and research, is defined as depression that develops within the first year postpartum [1]. It consists of a combination of depressed mood, loss of interest, anhedonia, sleep and appetite disturbance, impaired concentration, psychomotor disturbance, fatigue, feelings of guilt or worthlessness, and suicidal thoughts; these symptoms continue for more than two weeks [2]. Additionally, its onset occurs within four weeks of delivery [2]. A history of mood disorder and anxiety is a risk factor for postpartum depression [3]. There is not only one cause of postpartum depression, rather there are multiple biological factors, including hormonal factors, genetics, and immune function may be a cause of postpartum depression [1]. The prevalence of postpartum depression varies in each country, from 5.00% to 26.32%, and there is a high prevalence rate in developing countries [4, 5]. Postpartum depression can lead to 5–20% of maternal mortality [6–8]; postpartum depression seriously affects the behavioural symptoms in children [9]. The present value of total lifetime costs of perinatal depression was £75,728 per woman in the UK [10]. This means that the prevention of postpartum depression is essential. However, postpartum women are less likely to access prevention and treatment of postpartum depression due to various barriers such as lack of time, stigma, and childcare issues [11, 12].

To overcome these barriers, digital health technologies have made remarkable progress, such as telemedicine and the use of short message services (SMS), phone calls, and video calls using smartphones. Recently, application (app) -based interventions for prevention have been suggested. WHO defined mHealth as the new horizon for health through the use of mobile technologies: 1. cellular phone – utilization; 2. Computers, handheld – utilization; 3. Telemedicine; 4. medical informatics; 5. technology transfer; and 6. data collection [13]. There is a systematic review and meta-analysis about mHealth apps; their meta-analysis indicated that mHealth intervention improved the Edinburgh Postnatal Depression Scale (EPDS) scores in the treatment group compared to the controls [14]. There is another systematic review and meta-analysis on mHealth apps concerning symptom reduction of maternal depression and/or anxiety, which concluded conversely that this did not improve symptoms [15]. The mHealth intervention is a possible tool used to prevent and treat postpartum depression, but there is still a need for verification.

There have been some systematic reviews and meta-analyses of apps targeting the treatment of patients with postpartum depression, but there is no systematic review and meta-analysis focusing on apps preventing postpartum depression. Therefore, we conducted a systematic review and meta-analysis focusing on whether psychosocial intervention apps are effective in preventing postpartum depression. We focused on psychosocial interventions that are well-established approaches for postpartum women. This included peer support, counselling, educational programs, social support, cognitive-behavioural therapy, motivational interviewing, supportive care, mindfulness, and more [1].

## Methods

We conducted a systematic review and meta-analysis according to the Preferred Reporting Items for systematic reviews and Meta-Analysis (PRISMA) guidelines [16].

### Inclusion criteria

We included all RCTs that evaluated the app, including one with an automated component for preventing postpartum depression by providing psychosocial interventions.

Psychosocial interventions are non-pharmacological interventions that focus on psychological and social aspects, such as peer support, counselling, educational programs, social support, cognitive-behavioural therapy, motivational interviewing, supportive care, mindfulness, and so on. Participants receive psychosocial intervention via the app for smartphones, tablets, and computers. The app includes an automated component; the app itself has a function of psychosocial intervention, and participants could voluntarily access the app anytime, anywhere. This study incorporated a combination of automated apps and human interventions (e.g. phone calls and other interventions). However, we excluded the apps that only offered communication services like phone calls, short message services, chat, and social networking. If the psychosocial interventions were delivered via communication, we excluded it. We included women from pregnancy to one year postpartum and excluded those already diagnosed with depression, taking antidepressants, or suffering from psychiatric disorders. Quasi-randomised trials were excluded.

Inclusion criteria of PICOS principles are as follows.

- P: women who are pregnant and at postpartum (until one year after giving birth)
- I: the app, including an automated component purported to prevent postpartum depression by providing psychosocial interventions
- C: regular care

### Search strategy

We conducted an initial article search on 26 March 2020 and an updated article search on 17 March 2023 using the same search strategy. We conducted an article search of the electronic databases of the Cochrane Central Register of Controlled Trials (CENTRAL), MEDLINE via Ovid, Scopus, PsycINFO, CINAHL, and ProQuest Dissertations & Theses A&I. Additionally, we searched the International Clinical Trials Platform Search Portal (ICTRP) and Clinical Trials. The following terms were used: [postpartum depression] [computer software, mobile applications, and computer-assisted therapy]. The search strategy and terms have been presented in Supplementary table 1.

References cited in the identified randomized controlled trials (RCTs) and recent systematic reviews were searched.

An additional table file shows this in more detail (see Additional file named Supplementary table 1).

### Study selection, data extraction, and risk of bias assessment

There were five reviewers AS, KJ, TA, YO, and YM; pairs comprising these reviewers screened the titles and abstracts of the identified studies. All selected studies were subjected to a full-text review and evaluated based on the inclusion criteria by two reviewers (KK and YM).

Upon disagreement, the two reviewers discussed and resolved the issues. If any disagreements remained, they were resolved via discussion with a third reviewer (YO).

After the full-text review, if there were no sufficient data to decide whether the study should be included, we contacted the original author to decide about the final inclusion.

Two reviewers (KK and YM, TA and YM) independently assessed the quality of each study using the Cochrane Risk of Bias for RCTs. We evaluated random sequence generation, allocation concealment, blinding of participants and personnel, blinding of outcome assessments, incomplete outcome data, and selective outcome reporting to rate studies as ‘low risk’, ‘high risk’, or ‘unclear’. Upon disagreement, the two reviewers discussed and resolved the issues. If any disagreements remained, they were resolved via discussion with a third reviewer (YO).

### Types of outcome measures

The primary outcome was postpartum depression onset. Each study defined the onset of postpartum depression by using a clinical diagnostic interview or screening tool. A clinical diagnostic interview for depression is an evaluation conducted by a trained examiner based on an official diagnostic system, such as the Diagnostic and Statistical Manual of Mental Disorders fifth edition (DSM-5) [2], the International Classification of Disease (ICD-10), or other standard methods such as the Research Diagnostic Criteria (RDC) [17].

Secondary outcomes were scores on the EPDS [18], the Patient Health Questionnaire-9 (PHQ-9) [19], and other depression-related measures (e.g. the Centre for Epidemiologic Studies Depression Scale (CES-D) [20]). These scales have been validated for postpartum use [21–23]

### Data synthesis and statistical analysis

A random-effects model for the meta-analyses [24] was used, as there was clinical heterogeneity in the interventions explored in this review. Binary variables were calculated using risk ratios (RR) with 95% confidence intervals (95% CIs). Continuous variables were calculated using standardised mean differences (SMDs) with 95% CIs.

If there were missing data, we contacted the original authors whether we could get the data.

Heterogeneity was visually assessed using forest plots. I-square statistics [24], chi-squared statistics, and their *P* values were used to measure statistical heterogeneity. Data analysis was performed using Review Manager Version 5.4 (Nordic Cochrane Center, Cochrane Collaboration; Copenhagen, Denmark; http://ims.cochrane.org/revman).

## Results

**Fig. 1:**
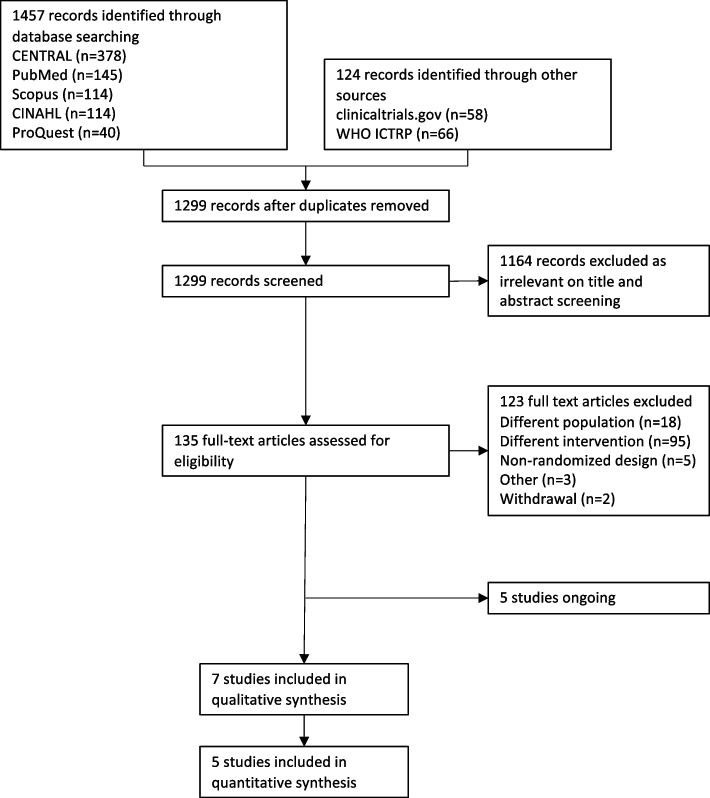
Study flow diagram of initial search on March 26.^th^, 2020

**Fig. 2:**
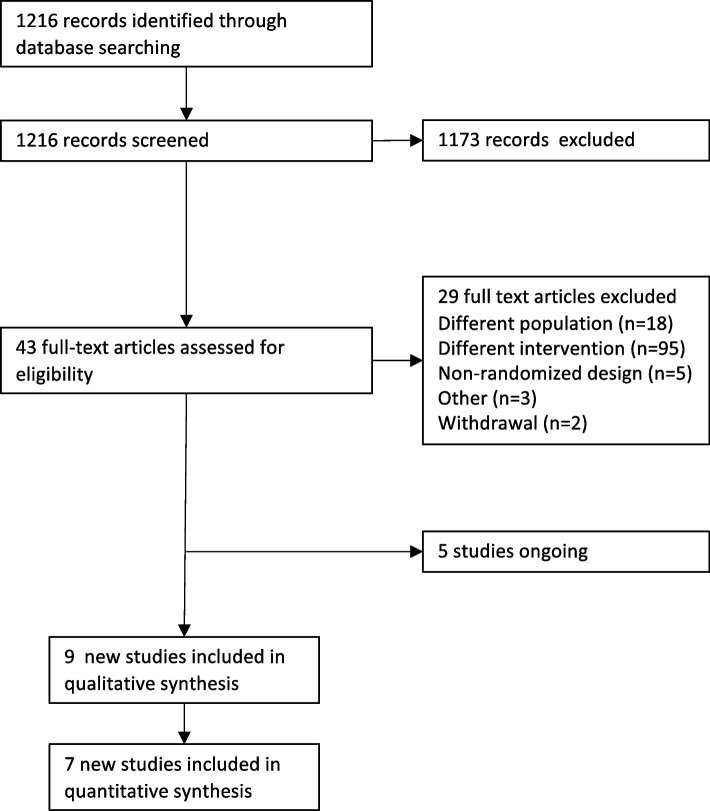
Study flow diagram of updated search on March 17.^th^, 2023

We conducted an initial search up to 26 March 2020. Figure 1 shows the flow diagram of the study selection process. We identified 1581 references and removed 282 duplicates. The titles and abstracts were evaluated, and 135 references were included in the full-text review. Hundred and twenty-three references were excluded because they did not meet the eligibility criteria, five were ongoing at the time, and seven were ultimately included in this review. In the updated search on 17 March 2023, we retrieved an additional 1216 references and found nine new studies included in a systematic review and five new ongoing studies (Fig. 2).

### Characteristics of included studies

Supplementary table 2 presents the characteristics of the studies included. All studies were published after 2015. There were three studies, each published in China, Portugal, and Singapore; two studies were published in the USA; one in Japan, New Zealand, Norway, Taiwan, and Turkey. In eight studies, the intervention started prenatally [25–32] and in another eight, the intervention started postpartum [33–40]. In three studies, the participants were married couples [28, 31, 33]. Two studies provided results only for couples [31, 33] and another solely for women [28].

There were six studies that provided interventions based on cognitive-behavioural therapy [25, 27, 30, 34, 38, 39], three that provided interventions based on mindfulness [29, 35, 36], one that provided interventions based on social cognitive theory and attachment theory [31], and another three that provided psycho-educational content [28, 32, 33]. The other three studies provided education or information about childcare [26, 37, 40].

Three studies assessed the development of postpartum depression [30, 32]. Fourteen studies assessed the EPDS. There was one study which evaluated the CES-D [27], and one study which evaluated the Depression Anxiety and Stress Scale 21 produces subscales of Depression (DASS-D) [35].

An additional table file shows this in more detail (see Additional file named Supplementary table 2).

### Risk of bias in the included studies

**Fig. 3:**
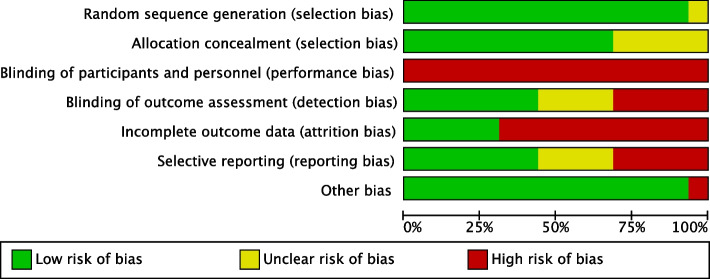
Risk of bias graph about primary outcome; the onset of postpartum depression

**Fig. 4:**
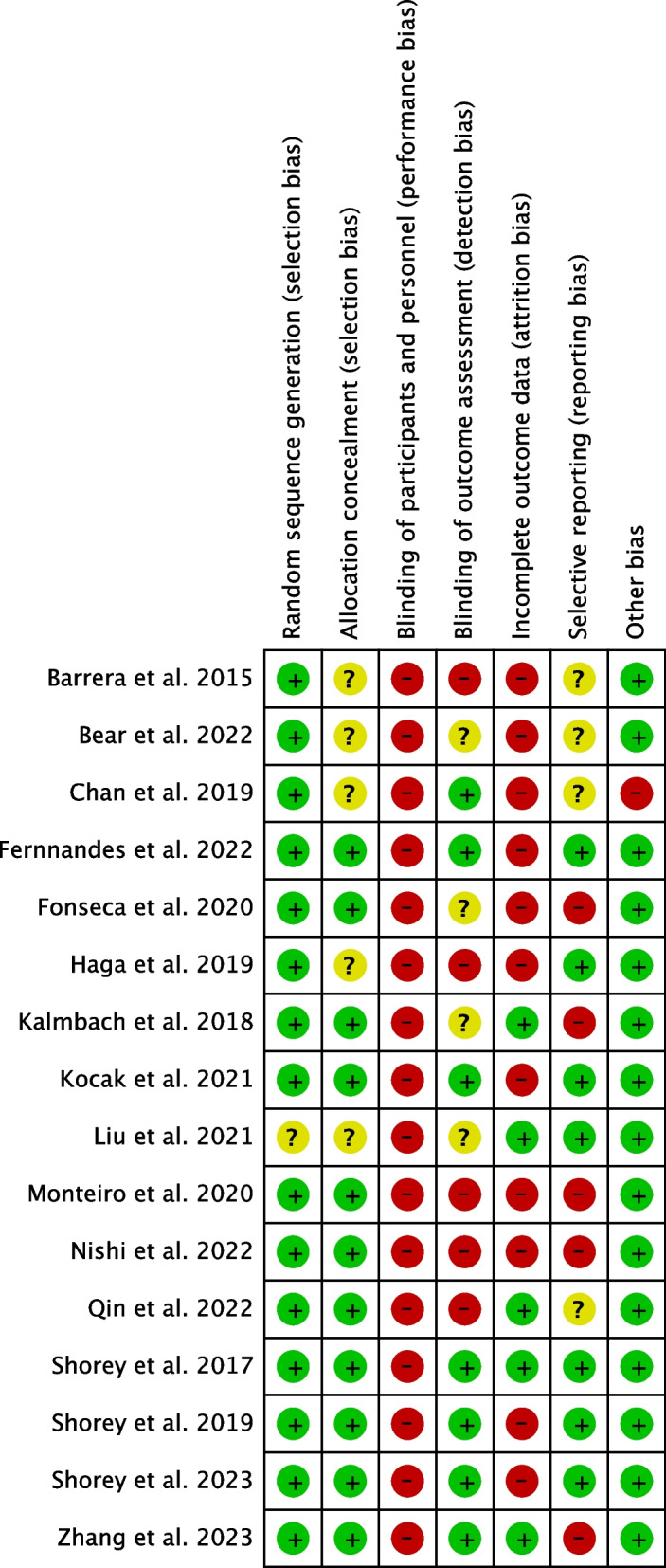
Risk of bias summary about primary outcome; the onset of postpartum depression

The risk of bias graph is shown in Fig. 3, and the risk of bias summary is shown in Fig. 4. All studies except one [29] were judged to have a low risk of bias about random sequence allocation. Allocation concealment was considered at low risk of bias in eleven studies and unclear in five studies [26, 27, 29, 32, 35]. App-based psychosocial interventions are difficult to blind, and all studies rated the risk of bias in blinding participants and personnel as high. Blinding of outcome assessment was judged to be at high risk of bias in five studies [27, 30, 32, 38, 39], unclear in four studies [25, 29, 34, 35], and low in seven studies. Incomplete outcome data were at high risk of bias in eleven studies [26–28, 30–32, 34–38] and low risk of bias in five studies. Selective reporting bias was judged to be at high risk of bias in five studies [25, 30, 34, 38, 40], unclear in four studies [26, 27, 35, 39], and low in eight studies. Other biases were at high risk of bias in one study [26] and low risk of bias in the other fifteen studies.

### Effects of interventions

#### Primary outcomes

**Fig. 5:**
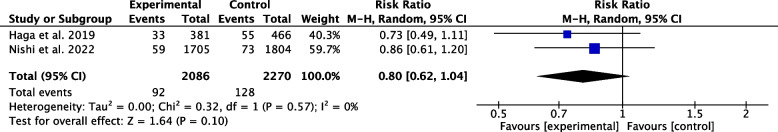
Effectiveness for the onset of postpartum depression

We conducted a meta-analysis of two studies on the onset of postpartum depression. There was no significant difference between the intervention and control groups (RR 0.80; 95%CI 0.62 to 1.04; *P* = 0.570; Fig. 5).

There were three studies assessed the onset of postpartum depression as an outcome [27, 30, 32].

Haga et al. defined an EPDS score of 10 or more as the onset of postpartum depression. Although EPDS was the screening tool, if authors defined an EPDS score of 10 or more as the onset of postpartum depression, we followed their definition.

 Nishi et al. also assessed the onset of postpartum depression based on the DSM-4 criteria.

Barrera et al. measured the EPDS score and calculated the hazard ratio (HR) using EPDS > 10 as the cut-off [27]. There was no statistically significant difference in the incidence of postpartum depression (EPDS score ≥ 10) between the intervention and comparison groups (HR 0. 60; 95%CI 0.34 to 1.02; *P* = 0.060). We could not get the number of participants who developed postpartum depression, so we did not include the study in the meta-analysis.

#### Secondary outcomes

There were fourteen studies that measured EPDS scores. Two of these fourteen studies include couples as participants, and we could not get outcomes about mothers [31, 33].

**Fig. 6:**
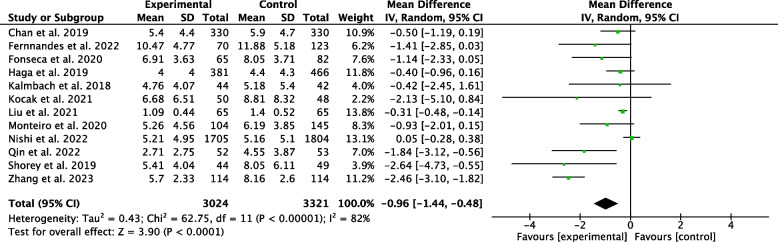
Effectiveness for EPDS score

We performed a meta-analysis of the EPDS scores of twelve studies. The intervention group had significantly lower EPDS scores than the control group (mean difference (MD) -0.96; 95%CI -1.44 to -0.48; *P* < 0.001; Fig. 6). There was high heterogeneity in the effects (I2 = 82.00%, Chi2 = 62.75, *P* < 0.001).

Bear et al. measured the DASS-D, and the linear regression model was tested with intervention vs. control as a predictor variable and outcomes. The intervention demonstrated a large effect on DASS-D, accounting for 48.00% of the variance [35].

Shorey et al. included couples as the participants in studies of 2017 [33]. There were no statistically significant differences in the EPDS scores between the intervention and control group (MD, 0.33; 95%CI -1.21 to 0.53; *P* = 0.450).

Shorey et al. published other studies about a psychoeducational mobile app for parents in 2023, there was no significant difference in the EPDS scores between interventions and control group (MD, 0.00; 95%CI -1.61 to 1.61; *P* = 1.000) [31].

PHQ-9 was not measured in either study.

Tolerability is an important outcome, but no studies evaluated it.

## Discussion

This systematic review and meta-analysis examined the effectiveness of the apps, including automated components aimed at preventing postpartum depression by providing psychosocial interventions. We included sixteen RCTs in the final analysis and conducted a meta-analysis of two studies on the onset of postpartum depression. App interventions may lead to the prevention of postpartum depression, but the results of the meta-analysis did not show a statistically significant difference with respect to the incidence of postpartum depression. However, the point estimate of the RR for those with postpartum depression was 0.80 and the 95%CI was not narrow enough to suggest a possible beneficial effect of the app intervention.

We also conducted a meta-analysis of the EPDS scores in twelve studies. The meta-analysis showed significantly lower EPDS scores in the intervention group, but there was high heterogeneity. There is the possibility that applications, which include one with an automated component for preventing postpartum depression via psychosocial interventions, may be effective to reduce the EPDS scores of postpartum mothers.

The recent coronavirus disease-19 (COVID-19) pandemic has had various impacts on pregnant and postpartum women, including fear of COVID-19, fear of infection, lifestyle changes due to lockdown, and social isolation. The decrease in physical activity during the COVID-19 pandemic has increased postpartum depression [41]. Indeed, the COVID-19 pandemic increased the prevalence of postpartum depression to 34.00% (95% CI 24.00–46.00%) [42], although the prevalence of postpartum depression was 20.80% (95% CI 17.90–33.80%) in middle-income countries and 25.80% (95% CI 18.40–23.10%) in low-income countries until 2017 [43]. The COVID-19 pandemic has forced us to limit face-to-face interventions for postpartum depression despite their increasing demand. This is a barrier for interventions to prevent postpartum depression, but app-based interventions that include an automated component can be a method that can overcome this barrier. Pregnant and postpartum women can use their smartphones or tablets to voluntarily engage in postpartum depression prevention, rather than face-to-face. After the COVID-19 pandemic, postpartum depression interventions with apps that include an automated component will receive more attention and tilization.

This study had some limitations. We conducted the meta-analysis of only two RCTs on postpartum depression onset [30, 32]. There was a variety of applications, some applications were based on cognitive behavioural therapy, others were based on mindfulness, and others on psychoeducation. Additionally, there was a high risk of incomplete outcome data due to the high attrition rates in the study.

## Conclusions

This study presents the results of current RCTs on interventions with apps, including an app with an automated psychosocial component for preventing postpartum depression that has been conducted. These apps improved the EPDS score; furthermore, they may prevent postpartum depression. There were many RCTs, that evaluated the reduction of postpartum depression symptoms, but few RCTs had evaluated the onset of postpartum depression. To see the effect of the apps in the prevention of postpartum depression, additional RCTs evaluating the onset of postpartum depression will be needed. 

## Supplementary Information

**Additional file 1: Supplementary Table 1.** The search strategies and terms.

**Additional file 2: Supplementary Table 2.** Characteristics of studies included in systematic review.

## Data Availability

All data analysed in this study are included within the article and list of references.

## Abbreviations

95% CI: 95% Confidence Intervals
App: Application
CENTRAL: The Cochrane Central Register of Controlled Trials
CES-D: The Center for Epidemiologic Studies Depression Scale
COVID-19: Coronavirus disease-19
DASS-D: The Depression Anxiety and Stress Scale 21 produces subscales of Depression
DSM-4: The Diagnostic and Statistical Manual of Mental Disorders, fourth edition, text revision
DSM-5: The Diagnostic and Statistical Manual of Mental Disorders fifth edition
EPDS: The Edinburgh Postnatal Depression Scale
HR: Hazard Ratio
ICD-10: The International Classification of Disease
ICTRP: International Clinical Trials Platform Search Portal
MD: Mean Difference
PHQ-9: The Patient Health Questionnaire-9
PRISMA: The Preferred Reporting Items for Systematic Reviews and Meta-Analysis
RCT: Randomized Controlled Trial
RDC: Research Diagnostic Criteria
RR: Risk Ration
SMD: Standardized Mean Difference
SMS: Short Message Service

## References

[1] Stewart DE, Vigod SN (2019). Postpartum Depression: Pathophysiology, Treatment, and Emerging Therapeutics. Annu Rev Med.

[2] Association AP (2022). Diagnostic and Statistical Manual of Mental Disorders, Fifth Edition (DSM-5).

[3] Wisner KL, Sit DK, McShea MC, Rizzo DM, Zoretich RA, Hughes CL (2013). Onset timing, thoughts of self-harm, and diagnoses in postpartum women with screen-positive depression findings. JAMA Psychiat.

[4] Liu X, Wang S, Wang G (2022). Prevalence and Risk Factors of Postpartum Depression in Women: A Systematic Review and Meta-analysis. J Clin Nurs.

[5] Shorey S, Chee CYI, Ng ED, Chan YH, Tam WWS, Chong YS (2018). Prevalence and incidence of postpartum depression among healthy mothers: A systematic review and meta-analysis. J Psychiatr Res.

[6] Jago CA, Singh SS, Moretti F (2020). Coronavirus Disease 2019 (COVID-19) and Pregnancy: Combating Isolation to Improve Outcomes. Obstet Gynecol.

[7] Grigoriadis S, Wilton AS, Kurdyak PA, Rhodes AE, VonderPorten EH, Levitt A (2017). Perinatal suicide in Ontario, Canada: a 15-year population-based study. CMAJ.

[8] Lindahl V, Pearson JL, Colpe L (2005). Prevalence of suicidality during pregnancy and the postpartum. Arch Womens Ment Health.

[9] Kingston D, Kehler H, Austin MP, Mughal MK, Wajid A, Vermeyden L (2018). Trajectories of maternal depressive symptoms during pregnancy and the first 12 months postpartum and child externalizing and internalizing behavior at three years. PLoS ONE.

[10] Bauer A, Knapp M, Parsonage M (2016). Lifetime costs of perinatal anxiety and depression. J Affect Disord.

[11] O'Mahen HA, Flynn HA (2008). Preferences and perceived barriers to treatment for depression during the perinatal period. J Womens Health (Larchmt).

[12] Goodman JH (2009). Women's attitudes, preferences, and perceived barriers to treatment for perinatal depression. Birth.

[13] eHealth WHOGOf (2011). mHealth: new horizons for health through mobile technologies: second global survey on eHealth.

[14] Zhou C, Hu H, Wang C, Zhu Z, Feng G, Xue J (2022). The effectiveness of mHealth interventions on postpartum depression: A systematic review and meta-analysis. J Telemed Telecare.

[15] Tsai Z, Kiss A, Nadeem S, Sidhom K, Owais S, Faltyn M (2022). Evaluating the effectiveness and quality of mobile applications for perinatal depression and anxiety: A systematic review and meta-analysis. J Affect Disord.

[16] Page MJ, McKenzie JE, Bossuyt PM, Boutron I, Hoffmann TC, Mulrow CD (2021). The PRISMA 2020 statement: an updated guideline for reporting systematic reviews. BMJ.

[17] Spitzer RL, Endicott J, Robins E (1978). Research diagnostic criteria: rationale and reliability. Arch Gen Psychiatry.

[18] Cox JL, Holden JM, Sagovsky R (1987). Detection of postnatal depression. Development of the 10-item Edinburgh Postnatal Depression Scale. Br J Psychiatry..

[19] Kroenke K, Spitzer RL, Williams JB (2001). The PHQ-9: validity of a brief depression severity measure. J Gen Intern Med.

[20] Radloff LS (1977). The CES-D Scale: A self-report depression scale for research in the general population. Appl Psychol Meas.

[21] Eberhard-Gran M, Eskild A, Tambs K, Opjordsmoen S, Samuelsen SO (2001). Review of validation studies of the Edinburgh Postnatal Depression Scale. Acta Psychiatr Scand.

[22] Wang L, Kroenke K, Stump TE, Monahan PO (2021). Screening for perinatal depression with the Patient Health Questionnaire depression scale (PHQ-9): A systematic review and meta-analysis. Gen Hosp Psychiatry.

[23] Heller HM, Draisma S, Honig A (2022). Construct validity and responsiveness of instruments measuring depression and anxiety in pregnancy: a comparison of EPDS, HADS-A and CES-D. Int J Environ Res Public Health.

[24] DerSimonian R, Laird N (1986). Meta-analysis in clinical trials. Control Clin Trials.

[25] Kalmbach DA, Cheng P, O'Brien LM, Swanson LM, Sangha R, Sen S (2020). A randomized controlled trial of digital cognitive behavioral therapy for insomnia in pregnant women. Sleep Med.

[26] Chan KL, Leung WC, Tiwari A, Or KL, Ip P (2019). Using Smartphone-Based Psychoeducation to Reduce Postnatal Depression Among First-Time Mothers: Randomized Controlled Trial. JMIR Mhealth Uhealth.

[27] Barrera AZ, Wickham RE, Muñoz RF (2015). Online prevention of postpartum depression for Spanish- and English-speaking pregnant women: A pilot randomized controlled trial. Internet Interv.

[28] Shorey S, Ng YPM, Ng ED, Siew AL, Mörelius E, Yoong J (2019). Effectiveness of a Technology-Based Supportive Educational Parenting Program on Parental Outcomes (Part 1): Randomized Controlled Trial. J Med Internet Res.

[29] Liu C, Chen H, Zhou F, Long Q, Wu K, Lo LM (2022). Positive intervention effect of mobile health application based on mindfulness and social support theory on postpartum depression symptoms of puerperae. BMC Womens Health.

[30] Nishi D, Imamura K, Watanabe K, Obikane E, Sasaki N, Yasuma N (2022). The preventive effect of internet-based cognitive behavioral therapy for prevention of depression during pregnancy and in the postpartum period (iPDP): a large scale randomized controlled trial. Psychiatry Clin Neurosci.

[31] Shorey S, Law E, Mathews J, Lim SH, Shi L, Chua JS (2023). Evaluating the Effectiveness of the Supportive Parenting App on Parental Outcomes: Randomized Controlled Trial. J Med Internet Res.

[32] Haga SM, Drozd F, Lisøy C, Wentzel-Larsen T, Slinning K (2019). Mamma Mia - A randomized controlled trial of an internet-based intervention for perinatal depression. Psychol Med.

[33] Shorey S, Lau Y, Dennis CL, Chan YS, Tam WWS, Chan YH (2017). A randomized-controlled trial to examine the effectiveness of the 'Home-but not Alone' mobile-health application educational programme on parental outcomes. J Adv Nurs.

[34] Fonseca A, Alves S, Monteiro F, Gorayeb R, Canavarro MC (2020). Be a Mom, a Web-Based Intervention to Prevent Postpartum Depression: Results From a Pilot Randomized Controlled Trial. Behav Ther.

[35] Bear KA, Barber CC, Medvedev ON (2022). The Impact of a Mindfulness App on Postnatal Distress. Mindfulness (N Y).

[36] Fernandes DV, Monteiro F, Canavarro MC, Moreira H (2022). A Web-Based, Mindful, and Compassionate Parenting Training for Mothers Experiencing Parenting Stress: Results from a Pilot Randomized Controlled Trial of the Mindful Moment Program. Mindfulness (N Y).

[37] Koçak V, Ege E, İyisoy MS (2021). The development of the postpartum mobile support application and the effect of the application on mothers' anxiety and depression symptoms. Arch Psychiatr Nurs.

[38] Monteiro F, Pereira M, Canavarro MC, Fonseca A (2020). Be a mom's efficacy in enhancing positive mental health among postpartum women presenting low risk for postpartum depression: results from a pilot randomized trial. Int J Environ Res Public Health.

[39] Qin X, Liu C, Zhu W, Chen Y, Wang Y (2022). Preventing postpartum depression in the early postpartum period using an app-based cognitive behavioral therapy program: a pilot randomized controlled study. Int J Environ Res Public Health.

[40] Zhang Y, Zhu J, Li S, Huang L, Fang Q, Zheng X (2023). The effectiveness of an internet-based support program on maternal self-efficacy, postpartum depression and social support for primiparous women during the COVID-19 pandemic: Randomized controlled trial. Front Public Health.

[41] Davenport MH, Meyer S, Meah VL, Strynadka MC, Khurana R (2020). Moms Are Not OK: COVID-19 and Maternal Mental Health. Front Glob Womens Health.

[42] Chen Q, Li W, Xiong J, Zheng X (2022). Prevalence and risk factors associated with postpartum depression during the COVID-19 pandemic: a literature review and meta-analysis. Int J Environ Res Public Health.

[43] Dadi AF, Miller ER, Mwanri L (2020). Postnatal depression and its association with adverse infant health outcomes in low- and middle-income countries: a systematic review and meta-analysis. BMC Pregnancy Childbirth.

